# Supplementary material for: Are carbon emissions trading and green financial instruments synergistic? -Comprehensive quantitative research based on content analysis
Source: PLoS One. 2024 Mar 7;19(3):e0298601. doi: 10.1371/journal.pone.0298601 (PMC10919601; doi:10.1371/journal.pone.0298601)
Supplement: S1 Data — (ZIP) [file pone.0298601.s001.zip › Data/地级市Z_F工作报告.zip!/╜¡╬≈/╨┬╙α╩╨/└┤╫╘╣1⁄2╓┌║┼í╛┬φ┐╦╩2╛▌═°í┐_2016.docx]

各位代表：

　　现在，我代表市人民政府向大会报告政府工作，并对“十三五”规划《纲要（草案）》作说明，请予审议，并请各位市政协委员和其他列席会议的同志提出意见。

　　一、2015年工作回顾

　　2015年，面对宏观经济依然疲软的大环境，在省委、省政府和市委的坚强领导下，全市上下坚持稳中求进工作总基调，牢牢把握经济社会发展主动权，主动适应经济发展新常态，克服了前所未有的困难，应对了重大风险的挑战，全力推进产业培育、项目建设、改革创新等工作，全市经济运行总体平稳、稳中有进、稳中有好，社会事业全面进步，人民群众获得感和满意度不断提升。预计全年完成生产总值955亿元，增长8.5%；实现财政总收入132.9亿元，增长5.1%，其中，公共财政预算收入98.8亿元，增长9.9%；规模以上工业增加值320亿元，增长7.6%；固定资产投资822.6亿元，增长10%；社会消费品零售总额207.3亿元，增长8.5%；出口总额78.7亿元，增长1.3%；实际利用外资3.7亿美元，增长5.8%，引进内资709.8亿元，增长14.3%；城镇居民人均可支配收入29836元，增长8%，农村居民人均可支配收入13986元，增长9%；居民消费价格指数上升1%；城镇登记失业率控制在3.7%。

　　㈠突出投资带动作用，项目建设成效显著。重点项目建设迈出新步伐。强化日常调度，创新协调机制，坚持每季度在县区流动召开项目建设推进会。建设重点项目70个，完成投资65亿元。出台《开展政府和社会资本合作的意见》，推出两批次共20个PPP项目，总投资26.3亿元，其中5个项目已确定社会资本方，3个签约项目在建。中电投分电扩建、大唐新电扩建、蒙华铁路新余段、沃格光电、木林森LED照明等五个市重大项目有序推进。招商引资工作取得新进展。坚持“建链、补链、强链”招商思路，市（县、区）主要领导每月带队外出招商，建立37支专业招商队伍，强力推进招商引资工作。共举办各类招商活动32次，实际引进省外5000万元以上项目资金377.8亿元，增长14.3%；签约“民企入赣”项目106个，总投资391.5亿元。政策资金争取实现新突破。强化政策研究，加大项目对接，共争取上级资金50.7亿元。节能减排财政政策综合示范城市工作三年总考评全国优秀，获国家奖励资金2.4亿元。资源枯竭城市转型年度绩效评价获全国第八，扶持政策成功延期，年度获中央转移支付资金2.7亿元。城市矿产示范基地实施方案调整通过国家部委批复。

　　㈡聚焦经济提质增效，转型升级步伐加快。工业结构持续调优。改造提升传统产业，扶持发展战略性新兴产业，推动工业经济加快结构调整。先后出台了促进经济稳增长10条意见，支持大众创业万众创新30条举措，减免工业项目规费10条措施，扶持装备制造业、房地产业和矿产资源企业发展办法等系列配套政策。安排产业引导基金2000万元、贷款贴息1000万元支持重点企业发展和项目建设。新兴产业加速发展，光电信息、装备制造、麻纺等产业增加值分别增长35%、20%、18%。光伏并网发电项目竣工137兆瓦，在建181兆瓦。安排倒贷周转金2.42亿元，帮助企业倒贷269笔合计40.5亿元。市国信担保公司在保企业156户，在保余额23.1亿元、增长20.8%。新增规模以上工业企业53户。赛维、江锂公司重组工作启动。成功举办国际麻纺博览会、麻纺产品设计大赛、南方农业（工程）机械展销会。第三产业不断壮大。大力发展新型业态，第三产业实现增加值365亿元，增长9.7%。新增规模以上服务业企业25户。电子商务实现交易额161亿元，增长56.3%。高新区电子商务产业园列为国家级示范基地。实现旅游总收入115.3亿元，增长27.2%。旅游发展总规修编完成，被授予“中国七仙女传说之乡”称号，昌坊度假村列为国家4A级旅游景区，中国洞都、亚热带植物园等投资5亿元以上景点开业。各项融资余额1400.7亿元，增长8.1%，其中贷款余额668.7亿元，增长11%。发放财园通、出口通、农贷通、科贷通贷款14.7亿元。沃格光电、金锂科技成功在“新三板”挂牌，增鑫牧业、百乐米业挂牌申请材料已获受理。九江银行新余分行、渝水湘淮村镇银行成功开业，兴业银行新余支行、分宜九银村镇银行启动筹建。文化产业实现增加值30亿元，增长25.7%。仙女湖国际汽车文化产业园开工建设。科技创新力度加大。以科技协同创新“六大引领工程”为抓手，不断发挥科技创新引领作用。5个项目获省科学技术奖。获批省级科技项目43个、资金636万元。新增高新技术企业20家。专利申请量1635件、授权量1068件，分别增长61.9%和63.8%；每万人有效发明专利量1.75件，增长59.1%。入选国家、省百千万人才工程分别新增2人和3人，国家级、省级博士后工作站分别新增2家和4家。

　　㈢注重统筹协调发展，城乡面貌持续改善。城市功能不断完善。通过健全规划体系、加快基础建设、推进精细管理，城市品位不断提升。城市总规实施情况评估初步完成，北湖、新钢、城南三个片区详规完成专家评审，消防、电力、城市照明等专项规划已报或通过省级审查。长青北路标准化改造完成，滨江路西延段、纵三路、五金路等建成通车。完成19条背街小巷和10个老旧小区基础设施改造、20个小区物业管理标准化建设。新建公租房1485套，发放租赁补贴6000户，改造棚户区5885户，新钢铁山棚户区改造完成，路东棚户区改造启动。建设改造农贸市场7个，新增净菜超市36个。完成排水管网缺陷修复30公里。廖家江改道工程扎实推进，贯早江排水渠改造完成。生活垃圾焚烧发电项目并网运行。镇村联动顺利推进。罗坊、双林、良山、河下四个中心镇污水处理设施基本建成，19个中心示范村“七改三网”和190个新农村点建设任务全面完成。“村组保洁、乡镇收集、县区转运、市级处理”的农村垃圾无害化处理模式确立，乡镇垃圾中转站基本建成。洞宜线改造、欧双公路改建完工，完成石镇线、上吉线、樟排线路面大中修65.8公里，改造县道21.1公里，建成通自然村水泥路100公里。新余至欧里、观巢城乡公交开通。小农水重点县、中小河流治理及水系连通等项目建设稳步推进，36座病险水库除险加固工程全部开工。现代农业加快发展。着力推进农业结构调整，全面激发农业发展活力，努力推动农业增效、农民增收。耕地保护责任全面落实，新增50亩以上种粮大户320家。特色农业发展加快，新余蜜桔、高产油茶种植面积分别达14.1万亩、18万亩，麒麟西瓜、无公害葡萄等产销两旺。生态循环农业势头良好，罗坊农业有机废弃物综合利用模式成为全省样板。新增农民合作社183家、家庭农场126家。省级以上农业产业化龙头企业实现销售收入118.6亿元。生态环境明显改善。强化绿色发展导向，出台了《关于建设生态文明先行示范区的实施意见》。国家和省35个重点减排项目如期建成，新钢、分电、新电和海螺等重点企业污染治理项目建成投运。深入开展货车超限超载集中整治和城市扬尘、燃煤锅炉、餐饮油烟等污染整治，空气质量优良天数为343天，优良率达94%。全国水生态文明城市建设试点工作有序推进。创建全国绿化模范城市通过检查验收，新增造林5.4万亩，森林抚育6.3万亩。“五山”保护利用取得新进展。分宜县被评为国家园林县城，双林镇被评为国家园林城镇。

　　㈣围绕增强发展活力，改革创新不断深化。以全面深化改革引领新常态，着力破解制约发展的体制机制障碍，28项改革任务全面完成。政府职能加快转变。“三单一网”改革深入推进，责任清单、权力清单全部公布。工商、质监、食药监实现“三局合一”，卫生、计生部门顺利合并，不动产登记职责完成整合。深化公共资源交易体制改革，建设工程招投标、政府采购、产权交易、国土资源交易四个要素市场实现整合。发布了政府购买服务指导目录，年度实施项目40个、资金9000万元。建立政府法律顾问制度，实现市、县（区）、乡（镇）三级政府法律顾问全覆盖。36个单位实行预算绩效目标管理试点。深入开展“三严三实”专题教育和“五大活动”，干部作风建设取得新成效。服务体系更加完善。新余政务服务网上线运行。408个行政村全部建立民事代办服务点。在全省率先建成社区数字化综合服务管理平台，便民办事系统在10个社区试运行，纳入事项123个，顺利实现市、区、街道和社区四级联办；公众服务系统“新余一网通”门户及手机APP上线试运行；社区网格化管理系统平台开发完成。市场活力加速释放。出台了社会信用体系建设规划，企业信用与金融服务一体化平台成功运行，新完成700余家企业信用等级评定，机关事业单位和公职人员信用体系建设工作启动。完成市属投融资企业改革重组，整合成立了市投资控股集团有限公司。探索金融助推实体经济发展新途径，组建了中科创业投资基金公司，市民间借贷登记服务中心建设方案确定。全面推行“三证合一、一照一码”商事制度改革，新登记企业7993户，新增注册资本405.8亿元。农村土地承包经营权确权登记颁证工作基本完成，新增农地流转面积3.4万亩，流转率达38.2%。

　　㈤紧扣改善民生福祉，社会事业全面进步。坚持把增进民生福祉作为最大责任，持续加大民生投入，30件实事全面完成。就业创业成效明显。新增城镇就业2.5万人，转移农村劳动力1.6万名。新发放小额担保贷款3.95亿元，扶持和带动1.3万人次就业创业。为工业园区定向培训1.6万人，创业培训3722人。社会保障能力加强。城镇居民医保年度最高支付额由9万元提高到10万元。新农合年人均筹资标准由390元提高到470元、参合率达99.2%，新农合大病保险实现全覆盖。城乡居民基础养老金每人每月由55元提高到80元。企业退休人员基本养老金十一连增。失业保险金发放标准每人每月由710元提高到950元。为各类困难对象发放保障救助资金2亿元。“十二五”35个贫困村如期实现脱贫。市养老福利中心一期工程竣工，新建社区居家养老服务中心14个、农村颐养之家46个。公共事业不断发展。严格规范招生秩序，全面停止小学阶段招收低龄生、义务教育阶段招收借读生、高中阶段招收择校生。在省级以上素质教育和职业技能竞赛中获奖344项（次）。明志、通济等5所小学扩容改造完成，新增学位7200个。校园周边食品安全整治成效明显。国家公共文化服务体系示范区创建工作扎实推进，实现文化设施网络化全覆盖。城市公立医院改革深入推进，村卫生计生服务室标准化、人口健康信息综合管理平台建设进展顺利。加强计生特殊家庭帮扶，荣获全国创建幸福家庭活动示范市。成功举办首届环仙女湖国际马拉松赛。实现全民健身站点社区全覆盖，十五分钟健身圈基本形成。社会治理持续创新。获全国文明城市提名资格。“新余有爱”志愿活动蓬勃开展。新钢公司社会职能移交工作稳步推进。智慧动员建设成效初显，创建双拥模范城全国“七连冠”、全省“八连冠”通过检查验收。“六五”普法任务全面完成。智慧新余建设成效明显，法治新余和平安新余建设深入推进，国家安全人民防线、信访维稳工作进一步加强，没有发生较大及以上安全事故，没有发生重大群体性事件，没有发生重特大刑事案件，全市社会和谐稳定。统计、审计、海关、检验检疫、民族宗教、外事侨务、涉台事务、档案、地方志、机关事务、防灾减灾、地质找矿、妇女儿童、青少年、老龄、科普、气象、人防、邮政通讯、烟草、盐业、海事、民兵预备役、社科研究、红十字会人道援助和慈善、残疾人事业等工作取得新成绩。

　　各位代表！2015年，新余经济社会发展砥砺前行，在巨大的压力面前能取得这样的成绩，实属不易。这得益于中央、省委省政府和市委的坚强领导，得益于市人大的法律监督和市政协的民主监督，得益于社会各界和全市广大人民群众的积极作为。在此，我代表市人民政府，向全市广大工人、农民、知识分子、干部和历任老领导、老同志，向各民主党派、工商联、无党派和社会各界人士，向驻市人民解放军、武警官兵和公安干警，向驻市中央、省属单位，致以崇高的敬意！向所有关心、支持新余发展的同志们、朋友们，表示衷心的感谢！

　　各位代表！回顾一年来的工作，对照市八届人大六次会议确定的目标任务，规模以上工业增加值、出口总额、社会消费品零售总额等指标增幅未能实现预期目标，分别与目标增速相差3个、3.7个和3个百分点。在此，我代表市人民政府，向各位代表深表歉意，并诚恳接受代表们的批评。同时，我们也清醒地认识到，全市经济社会发展还面临不少困难和问题：实体经济困难重重，新兴产业规模不大；新的财税增长点不多，财政收支平衡压力较大；项目数量和质量有待提升，经济发展后劲不足；去库存、降成本、补短板任务艰巨，公共服务、社会保障、生态建设等离人民群众的期盼还有差距；经济风险和社会矛盾日益显现，社会治理难度加大；少数干部担当精神不强，优化发展环境任重道远。对此，我们一定认真对待，切实加以解决。

　　二、2016年工作任务

　　2016年，是全面建成小康社会决胜阶段的开局之年。政府工作的总体要求是：全面贯彻党的十八大、十八届三中、四中、五中全会和中央、全省经济工作会议精神，认真贯彻习近平总书记系列重要讲话精神，深入贯彻落实“五大发展”理念，适应经济发展新常态，坚持“发展至上、富民为先”总思路，坚持稳中求进工作总基调，创新驱动促升级，深化改革添活力，狠抓项目扩投资，转变作风抓落实，全力推动经济稳定增长、产业转型发展、生态不断优化、民生持续改善，打好攻坚战，打赢“翻身仗”，为“十三五”开好局、起好步。

　　全市经济社会发展的主要预期目标是：生产总值增长8.5%，财政总收入和公共财政预算收入均增长8%，规模以上工业增加值增长9%，固定资产投资增长12%，社会消费品零售总额增长11%，外贸出口总额增长2.5%，实际利用外资增长9%，引进内资增长12%，城镇居民人均可支配收入增长8.5%，农村居民人均可支配收入增长9%，居民消费价格指数控制在3%左右，城镇登记失业率控制在4.5%以内，节能减排完成省下达的计划任务。

　　各位代表！我们要紧紧围绕上述目标，善于观大局、集中抓大事，坚持提速进位与转型升级并重，在努力克服经济下行压力中保持平稳较快发展，不断做优做强实体经济；坚持扩大投资与优化服务并重，在努力推进项目建设中打牢发展根基，不断增强经济发展后劲；坚持城乡建设与产业培育并重，在努力推进统筹发展中缩小城乡差距，不断提高城乡发展质量；坚持污染防治与生态保护并重，在努力推进绿色发展中加快建设美丽新余，不断提升生态文明水平；坚持深化改革与改进作风并重，在努力转变政府职能中优化创新创业环境，不断激发社会发展活力；坚持社会保障与公共服务并重，在努力推进社会事业全面发展中补齐民生民计短板，不断增进人民福祉。面对经济新常态、发展新任务、群众新期盼，打好“翻身仗”责任重大、任务艰巨。我们将团结和带领全市人民，进一步增强打赢“翻身仗”的信心和决心，进一步提振打赢“翻身仗”的精气神，进一步汇聚打赢“翻身仗”的强大正能量。重点做好以下工作：

　　（一）转变方式调结构，推动产业升级开创新局面

　　着力构建新型工业体系。推动主导产业转型发展。以供给侧结构性改革为导向，加快淘汰落后工艺、过剩产能和“僵尸企业”，加大钢铁、新能源、新材料三大产业技术改造力度，提升产品技术含量和附加值，推动主导产业向中高端迈进。稳妥推进赛维、江锂等企业重组，加快引进战略合作伙伴，助推企业走出困境。培育壮大新兴产业。光电信息产业重点向终端产品延伸，加快质量检测、科技研发等公共平台建设，确保主营业务收入突破200亿元、增长90%。装备制造业以汽车零部件为主导，大力发展农用机械、工程机械等产品，确保主营业务收入突破120亿元、增长20%。积极争取国家光伏发电“领跑者计划”，探索“农光”“林光”“渔光”互补发展新模式，确保光伏并网发电项目新增220兆瓦。加快发展食品医药、节能环保、苎麻纺织等产业，着力培育新的经济增长点。全面提升园区竞争优势。加快盘活土地和厂房等资源，完善配套设施，新增标准厂房35万平方米，实现规模以上企业主营业务收入1200亿元、增长10%以上。大力推进高新区标准化园区暨创新创业孵化基地建设，力争实现规模以上企业主营业务收入810亿元、增长14.5%。切实抓好企业帮扶。出台更加有力的政策举措，帮助企业降低制度性交易成本、税费负担、财务成本、物流成本等，提升企业市场竞争力。创新企业帮扶机制，遵循市场规则和法治原则，加强政策引导和服务，将有限的资源配置到优质企业和成长性产业上，确保新增规模以上工业企业55户以上。

　　全力提升第三产业比重。强化服务业运行调度，努力提高第三产业占生产总值的比重，新增规模以上服务业企业20户。推进大型商贸综合体、商业功能区、特色商业街区建设，推动城市商业提档升级。统筹推进跨境电商、社区电商和农村电商发展，建设集电商、仓储、配送于一体的现代化综合物流园区，力争实现电商交易额260亿元、增长62%以上。加快发展大众化餐饮业，着力打造特色美食街区。大力发展会展经济，积极打造国际麻纺博览会、抱石文化节等会展品牌。加快推进仙女湖创建国家5A级景区工作，抓好智慧旅游城市建设，力争实现旅游总收入138亿元、增长20%。积极引进培育一批优势文化企业，力争新增规模以上文化企业7户。加快金融机构引进步伐，力争年末实现各项融资余额1520亿元、增长8.5%。支持企业上市融资和发行企业债券，力争5家以上企业在“新三板”挂牌，实现直接融资余额320亿元、增长16%以上；完善融资担保体系，充分运用财园通、出口通、农贷通、科贷通和产业发展基金，拓宽企业融资渠道。加强体育与旅游、文化的融合对接，办好环仙女湖国际马拉松和自行车大赛，大力培育体育消费市场。

　　充分发挥创新引领作用。以科技创新驱动产业结构调整，完善创新资源配置机制，确保高新技术产业增加值占规模以上工业增加值比重达25%。强化企业主体地位，确保有创新活动的规模以上企业占比达15%，新增高新技术企业10家以上。加强创新平台建设，培育市级工程中心5家、创新团队5家，申报省级以上科技项目超40个，实施企业产学研合作项目25个以上。力争每万人发明专利拥有量达1.8件，专利申请量1800件、授权量1000件。

　　（二）固本强基扩投资，推动项目建设实现新跨越

　　积极争取项目资金。坚持“管长远、增后劲、补短板”的原则，重点按照国家发改委关于加强政府投资项目储备、编制三年滚动投资计划的要求，认真谋划一批基础设施、工业经济、公共服务等领域重大项目。加大向上争资金、争项目、争政策力度，出台争取上级资金考核办法，力争争取上级资金增长10%以上。切实做好专项建设基金项目申报工作。确保资源枯竭城市转型、“城市矿产”示范基地等项目顺利通过国家考核和检查。

　　提高招商引资实效。坚持“既要数量，更要质量”的招商理念，强化经济部门招商引资责任，组建72支专业招商小分队，精心组织24场专题招商推介活动，构建市县（区）和部门联动招商新机制。大力开展以商招商、商（协）会招商，提高招商引资市场运作水平。围绕新能源、新材料、光电信息、装备制造、节能环保、文化旅游等产业，开展定向、定点、定单等专题招商，推动产业纵向链接、侧向配套，实现集群发展。

　　加快重点项目建设。紧盯重点项目数量和投资规模“两个翻番”目标，完善跟踪服务，实行分类调度，强化督查考核，确保重点项目达140个、年度投资130亿元。突出抓好中电投分电扩建、大唐新电扩建、蒙华铁路新余段等重大项目，重点推进木林森LED照明产品及配套、沃格光电玻璃精深加工、新联泰车轮钢圈一体化、恩达高档面料印花生产线等续建项目，加快沐阳手机外壳压铸、科陆电子逆变器、科利亚农业机械、金锂科技锂电池正极材料生产线等新建项目开工步伐，力促项目早建成、早投产、早见效。

　　（三）多措并举补短板，推动城乡统筹取得新成效

　　加快完善城市基础功能。立足打造“精美特新”中小城市典范，不断完善城市基础设施，补齐城市功能不全的短板，逐步解决“城市病”。加强中心城区城市设计，完成孔目江新城片区控规编制，抓好海绵城市系统、地下综合管廊等规划编制。两年内全面完成全市主要背街小巷改造和城中村、老旧小区基础设施的完善，今年集中精力完成城南片区的改造、完善工作。出台棚户区改造鼓励货币化安置政策，抓好新钢路东棚户区改造，完成吉泰苑保障房项目建设，启动新纺、西街棚户区改造工程。全面改造新欣大道、五一路、天工大道、天工大桥至环城南路，新建百乐路、塘下路、清新路、伴山路，启动果蔬批发市场跨孔目江大桥、阳光大道跨上新铁路桥建设，完善城区路网体系。抓好污水管网改造和排水管网隐患排查，全面完成廖家江改道工程，改造廖家江、贯早江水渠景观。建设公共自行车系统，推进公交站台和智能电子站牌建设改造，优化公交运行线路，提升群众出行便捷度和舒适度。强化政策引导，打通供需通道，消化库存，稳定房地产市场。促进建筑业发展，确保建安产值不低于120亿元。大力推进智慧城市建设，全面提高城市智能化、网格化、精细化管理水平。规范居民小区管理，努力提升物业管理和服务水平。

　　加快推进镇村联动建设。坚持以项目建设为抓手，巩固扩权强镇改革成果，重点推进罗坊、双林公共服务提升和良山、河下基础设施完善。突出抓好新农村点建设和中心示范村“六个重点”建设。深入开展农村生活垃圾专项治理，实现卫生保洁自然村全覆盖。建成欧里至东岳庙公路、人和至洞村连接线工程，完成袁河大桥危桥改建，建设通自然村水泥路100公里、公路安全生命防护工程200公里、农林场公路30公里，升级改造县乡道20公里、农村客运网络化公路24公里，改建农村公路危桥10座。新建和改造城市配电网线路104公里，推进农村中低压配电网升级改造，提高城乡供电稳定性。

　　加快转变农业发展方式。以现代化农业园区建设为抓手，加快农村土地流转，不断夯实现代农业发展基础。着力培育发展加工型农业产业化龙头企业，新增规模以上农业企业3户以上、销售收入超亿元企业1户以上。加强农产品销售平台建设，重点扶持新余蜜桔、高产油茶等特色产业发展，推动农产品精深加工和综合开发。加快发展集生态保护、农耕文化、休闲娱乐、乡村旅游和农事体验为一体的休闲农业。制订生态循环农业发展规划和政策扶持办法，大力推广罗坊农业有机废弃物综合利用模式。全面完成城市周边永久基本农田划定。抓好病险水库除险加固、中小河流综合整治及水系连通、农村饮水安全巩固提升等项目建设。

　　（四）持之以恒优环境，推动生态文明达到新水平

　　深入推进节能减排。抓好城西污水处理厂及管网建设，完善良山、下村、分宜三个污水处理厂管网，提升污水收集率，解决建成区污水直排问题。加强在线监控，确保钢铁、电力、水泥等重点行业脱硫、脱硝、除尘设施稳定运行。强化畜禽养殖场环境管理，完善污染减排和治理设施。大力推进民用建筑节能和绿色建筑发展。

　　从严实施环境保护。继续开展“净空”“净水”“净土”行动。加强城市扬尘控制和货车超限超载集中整治，加快淘汰黄标车、老旧机动车，完善餐饮业油烟防治措施，不断提升大气环境质量。创新河湖保护管理体制，全面建立和落实覆盖市、县（区）、乡（镇）的“河长制”。切实做好“五山”和后备水源地保护。加大环境执法力度，严惩违法违规行为。

　　加快建设美丽新余。积极融入全省生态文明先行示范区建设，走生态优先、绿色发展之路，实现百姓富、生态美的有机统一。新建北湖公园至仰天岗纪念碑广场城市绿轴、暨阳人民广场，加快北湖公园东出入口及配套设施工程建设，推进公园夜景、桥梁亮化、街头小游园及绿地提升改造。完成造林3万亩，森林抚育4万亩。以合同环境服务试点为抓手，鼓励各类投资主体参与生态环境建设、生态经济项目开发，培育发展壮大一批环保服务企业。

　　（五）坚定不移促改革，推动市场主体迸发新活力

　　深化行政管理体制改革。加快政府职能转变，推进政府管理由注重事先审批向注重事中服务、事后监管转变。建立审批事项动态清理工作机制，完善“三单一网”管理，推进审批流程优化再造，实现所有行政审批全程网上运行。进一步推进“先照后证”、市场主体住所登记等改革，加快推行电子营业执照和企业注册全程电子化。按照“成熟一个，实施一个”的原则，探索生产经营性事业单位转为企业或社会组织。加快中介组织与政府部门脱钩，进一步开放中介市场，形成市场竞争充分的中介服务环境。

　　深化经济体制机制改革。深入推进投融资体制改革，改进财政资金投资方式，采取PPP模式和发展风险投资、产业基金、私募基金等，引导更多社会资本参与各类项目建设。加快全口径预算制度改革，将非税收入纳入预算管理，全面推行预算绩效目标管理。完善国有资产管理体制，积极稳妥发展混合所有制经济。深化公共资源交易改革，探索逐步上收乡镇公共资源交易权限。加快农村集体产权制度改革，继续开展农村集体产权股份合作制度改革试点。

　　深化社会管理体制改革。进一步推进各项便民惠民制度改革，完善和推广社区数字化综合服务管理。推进信用体系平台建设，强化信用信息运用，加快信用新余建设进程。建立健全生态补偿机制，出台重点流域、森林等生态补偿办法。深化户籍制度改革，全面落实居住证制度。深入推进涉法涉诉和信访综合改革，构建畅通、规范、高效的信访工作新机制。推广政府购买服务模式，扩大社会组织提供公共服务范围。深化公立医院改革，推进医疗、医药、医保“三医”联动。

　　（六）综合施策惠民生，推动社会事业迈上新台阶

　　倾力改善民生福祉。坚持民本为先、民本为要、民本为重，始终把解决好群众最关心、最直接、最现实的利益问题放在首位，牢牢守住民生底线，千方百计筹集各类资金13.8亿元，围绕完善市政基础设施、改善群众生产生活条件、提升便民服务水平等方面，全力以赴办好40件民生实事，让全市人民出行更方便、办事更快捷、生活更舒适。认真落实推进大众创业万众创新的实施意见，多渠道帮助各类群体实现就业创业。进一步提高社会保障水平，把重点放在兜底上，保障群众基本生活。抓好市养老福利中心建设和乡镇敬老院新改扩建，加快构建以居家养老为基础、社区为依托、机构为支撑的覆盖城乡的多样化养老服务体系。提高社会救助工作水平，实现城乡低保应保尽保。围绕2017年底“十三五”贫困村全面脱贫目标，大力实施“光伏扶贫”工程，确保5.2兆瓦光伏发电扶贫项目并网发电。

　　着力建设平安新余。大力推进依法治市，全面加快科学立法、严格执法、公正司法、全民守法进程，着力打造法治新余。加强国家安全人民防线建设和公共安全应急、防暴、处突管理，加大“黄赌毒”综合治理力度，防范和打击各类违法犯罪。强化安全生产责任落实，预防重特大安全事故发生。高度重视食品安全，确保人民群众“舌尖上的安全”。

　　全力发展公共事业。扩充学前教育资源，加大义务教育学校校长、教师交流轮岗力度，推进教育信息化建设，鼓励社会力量和民间资本办学。支持新余学院转型发展，推进职业教育转型升级，深化现代学徒制试点，提高各类教育服务地方经济发展的水平。鼓励社会力量兴办规模化、特色化的医疗机构。认真落实全面两孩政策。抓好国家公共文化服务体系示范区创建，加快推出一批文化精品，广泛开展文化惠民活动，倡导全民阅读，打造书香新余。推进国家信息惠民和“宽带中国”示范城市建设，实施城乡全光网行动计划。以创建全国文明城市为契机，大力弘扬社会主义核心价值观，不断提升市民文明素质。广泛开展全民健身运动，办好第八届市运会。关心支持妇儿、青少年、老龄事业，发展慈善和残疾人事业。加强国防后备力量和人民防空建设，推进“双拥”和优抚安置。做好新形势下的科普、气象、史志、档案、外事侨务、民族宗教、驻外机构、防灾减灾、邮政通讯等工作。

　　三、关于《新余市国民经济和社会发展第十三个五年规划纲要(草案)》的说明

　　市委、市政府高度重视“十三五”规划编制工作，成立专门领导小组，多次召开专题会议，对事关新余发展的重大问题进行研究决策。市人大、市政协分别提出建议，为规划编制提供了重要支撑。根据市委七届十九次全会审议通过的《关于制定新余市国民经济和社会发展第十三个五年规划的建议》，在广泛听取各民主党派、工商联和无党派人士、专家学者、广大市民意见建议基础上，市政府制定了《新余市国民经济和社会发展第十三个五年规划纲要（草案）》，已提请大会审议。

　　《纲要（草案）》全文共十一篇三十八章。第一篇分析了“十三五”时期新余经济社会发展的基础和背景，提出了“十三五”规划的指导思想和发展目标，确立了“十三五”时期“提前全面建成小康社会”这一总目标；第二篇到第七篇提出了推进“六个新余”建设的主要任务；第八篇到第十一篇主要从加强基础设施建设、全面深化改革、全面依法治市和全面从严治党四个方面，提出了保障规划顺利实施的具体措施。下面，我作几点简要说明。

　　（一）“十二五”时期我市经济社会发展的主要成就

　　过去的五年，是新余发展史上极不平凡的五年。面对错综复杂的国内外发展环境和经济下行压力，在省委、省政府和市委的坚强领导下，全市人民凝心聚力、顽强拼搏、攻坚克难、开拓创新，不断开创各项事业发展新局面。特别是市委七届九次全会以来，坚定不移贯彻习近平总书记系列重要讲话精神，坚定不移落实省委“发展升级、小康提速、绿色崛起、实干兴赣”十六字方针，坚持发展至上、富民为先，主动适应经济发展新常态，统筹推进稳增长、促改革、调结构、惠民生、防风险、强作风各项工作，全市经济社会发展取得新成就。生产总值年均增长9%，三次产业比由6:63.9:30.1调整为5.7：55.8：38.5，财政总收入年均增长10.5%，规模以上工业增加值年均增长10.3%，全社会固定资产投资年均增长12.5%，社会消费品零售总额年均增长13%。统筹城乡建设成绩显著，主城区建成面积达70平方公里，城镇化率达68.2%；杭南长高铁和蒙华铁路新余段、中电投分电和大唐新电扩建等一批事关新余长远发展的项目相继开工或建成投运。改革开放深入推进，“小政府、大社会、活市场、优环境”四大改革工程全面推进，重点领域和关键环节改革取得重大突破；引进内资年均增长23.8%。人民生活水平稳步提高，城镇居民和农村居民人均可支配收入年均增长11.4%和13.3%，城乡收入差距进一步缩小。生态环境建设成效明显，“三项政策”顺利实施，被评为国家森林城市、国家园林城市。社会事业全面进步，科技、教育、文化、卫生等各项社会事业日益繁荣，社会治理体系不断完善，社会更加和谐稳定。

　　过去五年取得的成就，是市委、市政府认真贯彻中央和省各项方针政策，团结带领全市人民共同努力奋斗的结果。过去五年的发展历程，最根本的是把上级精神与新余实际紧密结合起来，不断增强工作的前瞻性、创造性，探索出一条符合科学发展要求、具有新余特色的发展道路。过去的五年，为“十三五”乃至更长时期的发展奠定了基础、积蓄了能量、增强了后劲。展望未来，新余已经站在了一个更高的历史起点上！

　　（二）“十三五”时期经济社会发展的主要目标

　　“十三五”时期，是我市迈向全面小康的决战期、经济转型升级的关键期、生态文明建设的提升期、全面深化改革的攻坚期、法治新余建设的推进期。《纲要（草案）》指导思想与市委《建议》提出的指导思想保持了高度一致。同时，根据市委《建议》，《纲要（草案）》围绕提前一年全面建成小康社会，在经济发展、转型升级、社会民生、生态文明等四方面，提出了“十三五”时期经济社会发展的36个主要目标，其中预期性指标16个、约束性指标20个。

　　这里，我就其中三个方面的重要目标作简要说明。

　　第一，关于奋斗目标。《纲要（草案）》提出提前一年全面建成小康社会目标，主要基于以下考虑：一是工作基础比较扎实。“十五”“十一五”“十二五”时期，我市小康社会实现程度年均分别提高了2.2、3.1和2.3个百分点。提前1年全面建成小康社会，在“十三五”时期前四年，每年仅需提高1.7个百分点。二是重点指标已有突破。全面建成小康社会的39项监测指标中，需重点突破的指标有9项，其中只有第三产业增加值占生产总值比重、文化及相关产业增加值占生产总值比重、单位生产总值能耗、主要污染物排放强度指数4项指标完成目标任务的压力较大。但经过近年来重点攻坚，均有较大突破。如第三产业增加值、文化及相关产业增加值占生产总值的比重，过去五年分别提高了8.2和1.6个百分点。三是发展趋势稳中向好。目前我市经济发展的基本面是好的，尤其是通过近三年持续不断地促改革、打基础，一些制约经济社会发展的体制机制障碍基本理顺，一些历史遗留问题有望在近期得到解决。一系列刺激经济发展的政策效应将逐步显现，经济触底反弹的局面有望实现。一批重大项目，特别是“两厂一路一基地”等项目的实施，将进一步拉动全市经济增长。光电信息、装备制造、光伏发电等新兴产业迅速崛起，对推动产业转型升级将起到积极促进作用。同时，通过开展党的群众路线教育实践活动、“三严三实”专题教育和“五大活动”，广大干部干事创业的精神面貌有了明显提升。上述有利因素，进一步增强了我们提前全面建成小康社会的信心和决心。

　　第二，关于经济增速。《纲要（草案）》提出“十三五”时期生产总值年均增长8.5%，主要考虑：一是与经济步入中高速增长的新常态相契合；二是实现提前一年全面建成小康社会，生产总值年均增速需达到7.2%以上；三是第三产业增加值占生产总值的比重等指标，是我市全面建成小康社会的“短板”指标。如果生产总值增速定得越高，“短板”指标完成目标任务的难度就越大。综合考虑以上三方面因素，生产总值增速设定为8.5%，是个适中的速度，符合新余实际。

　　第三，关于居民收入。《纲要（草案）》提出，城镇居民和农村居民人均可支配收入年均分别增长8%和9%，主要是考虑到确保居民收入增长与经济发展同步、缩小农村居民与城镇居民收入差距等因素。

　　（三）“十三五”时期经济社会发展的主要任务

　　《纲要（草案）》对“十三五”时期的经济发展、科技创新、城乡统筹、生态文明、改革开放、社会民生等方面进行了全面部署，主要是围绕建设“六个新余”，从方向和途径、定位和趋势上明确了今后五年的发展重点。

　　1.关于“实力新余”。《纲要（草案）》提出，要以新型工业化为核心，推进现代农业和现代服务业加快发展，推动产业结构转型升级，促进产业发展模式向低碳绿色转变、产业层次向中高端迈进，打造技术先进、协调融合、优质高效、绿色低碳的现代产业体系。

　　2.关于“智慧新余”。《纲要（草案）》提出，要以全面增强科技创新能力为核心，突出企业科技创新主体地位，推进重点领域创新与大众创业万众创新相结合，推动信息技术广泛渗透和运用，打造中小智慧城市样板。

　　3.关于“品质新余”。《纲要（草案）》明确，要以改革创新为统领，以城乡统筹为导向，以产城融合为抓手，大力推进“以人为核心”的新型城镇化，全面提升城镇化发展质量，加快构建新型城乡关系，加强社会主义精神文明建设，打造“精美特新”中小城市典范。

　　4.关于“美丽新余”。《纲要（草案）》提出，要紧紧抓住全省建设生态文明先行示范区的机遇，把生态文明建设融入经济社会发展各方面和全过程，加快形成绿色发展方式和生活方式，打造天蓝、地绿、水净的美丽新余。

　　5.关于“活力新余”。《纲要（草案）》提出，要牢牢把握发展战略机遇，坚持对内开放与对外开放并举、引资与引技引智结合、“引进来”与“走出去”并重，形成立体式、多循环开放大格局，打造开放型经济升级版。

　　6.关于“幸福新余”。《纲要（草案）》提出，要按照人人参与、人人尽力、人人享有的要求，坚持守住底线、突出重点、完善制度、引导预期，注重机会公平，保障基本民生，切实增强人民群众的幸福感和获得感。

　　需要特别说明的是，《纲要（草案）》在市委《建议》基础上，增加了“加强基础设施建设，构建经济社会发展新支撑”一篇。我们认为，未来几年我市经济社会发展的工作重点、难点和落脚点均在于此，如“两厂一路一基地”、光伏应用、城市大小外环、信息化网络建设等，都是实实在在的项目，如能顺利实施，必将有效解决制约我市经济社会发展的瓶颈问题，应予以高度重视，故单列一篇。

　　同时，《纲要（草案）》提出，要以经济体制改革为重点，正确处理政府、市场、社会的关系，努力在重点领域和关键环节改革上取得决定性成果，为经济社会发展提供持久动力和制度保障；要运用法治思维和法治方式推动发展，营造公平正义的法治环境与和谐稳定的社会环境，确保法治新余建设取得成效；要强化党的领导核心作用，切实履行政府职责，举全市之力、集全市之智，确保实现未来五年发展的宏伟蓝图。

　　各位代表！全面完成2016年目标任务，确保打赢“翻身仗”，对政府工作提出了新的更高要求。我们将坚持问题导向、市场导向、需求导向，切实加强政府自身建设，努力打造群众满意的人民政府。

　　我们要着力推进依法行政。健全依法决策机制，严格落实公众参与、专家论证、风险评估、合法性审查、集体讨论决定等重大行政决策程序，推进政府决策科学化、民主化、法治化。认真执行市人大及其常委会的决议和决定，自觉接受人大法律监督和工作监督。积极听取各民主党派、工商联、各人民团体和各界人士的意见建议，自觉接受政协民主监督。切实加强同人大代表和政协委员的联系，认真办理好建议提案。全力推进政务公开，主动接受司法监督、舆论监督和公众监督。

　　我们要切实加强廉政建设。认真贯彻落实“一准则、两条例”，严格执行党风廉政建设责任制，深入开展反腐败斗争，强化廉政风险防范管理，加强行政权力监督制约，严肃查处发生在群众身边的不正之风和腐败问题，切实做到清正廉洁、务实为民。扎实推进“违插”专项治理，严格落实土地出让、工程招标、政府采购等各项规定。大力弘扬勤俭节约的优良传统，进一步压缩“三公”经费，集中力量促发展、惠民生。

　　我们要不断改进工作作风。以“三严三实”的态度和决心，加快转变干部作风，不断增强服务经济社会发展的责任感和使命感。探索建立容错机制、考评机制和保护机制，旗帜鲜明地褒奖鼓励、支持保护担当者，营造敢于担当、善于担当的从政环境。着力解决“中梗阻”“为官不为”等作风问题，进一步优化发展环境。认真落实首问负责制、限时办结制、服务承诺制等制度，切实提高工作效能。完善科学合理的绩效考核体系，建设网上绩效考核评价系统，强化考核过程管理，确保政令畅通、令行禁止。牢固树立群众观念，感同身受群众困苦，通过一件件实事的办理、一件件难事的解决，赢得人民群众更多的信任和更大的支持。

　　各位代表！新余的发展迈上了新的历史征程，美好前景催人奋进。让我们更加紧密地团结在以习近平同志为总书记的党中央周围，在市委的坚强领导下，认识新常态、适应新常态、引领新常态，坚定信心、保持定力，奋发有为、真抓实干，为打赢“翻身仗”、实现“十三五”良好开局而努力奋斗！
